# Light People: Professor Laura Na Liu

**DOI:** 10.1038/s41377-023-01356-3

**Published:** 2024-01-23

**Authors:** Hui Wang, Cun Yu

**Affiliations:** Changchun Institute of Optics, Fine Mechanics and Physics, Chinese Academy of Sciences, 130033 Changchun, China

**Keywords:** Nanophotonics and plasmonics, Biophotonics

## Abstract

The DNA nanotechnology outlines a new chapter in biological sciences, opening a new era of scientific and technological advancements. A pioneer of DNA nanotechnology, Professor Liu from the University of Stuttgart in Germany, our latest Light People, has been working at the interface, where nanophotonics meets biology and chemistry. On the path of scientific explorations, she believes in hard working and persistency, as indicated by a line of Chinese poetry she likes, “Don’t stop chasing the wind and the moon, the spring mountain is at the end of the plain.” She is an outstanding female researcher, radiating wisdom. She is a pioneer in exploring the power of DNA nanotechnology and applying it to other disciplines. Meanwhile, she struggles with work–life balance. Apart from being determined and persistent, Prof. Liu is also introspective. One sees the brilliance of a scientist and the struggles of a mother, as Prof. Laura Na Liu explains to us her very personal “philosophy of life”.

## Abstract (web summary)

A pioneer of DNA nanotechnology, Prof. Laura Na Liu from the University of Stuttgart in Germany, has been working at the interface, where nanophotonics meets biology and chemistry.

## Graphical abstract

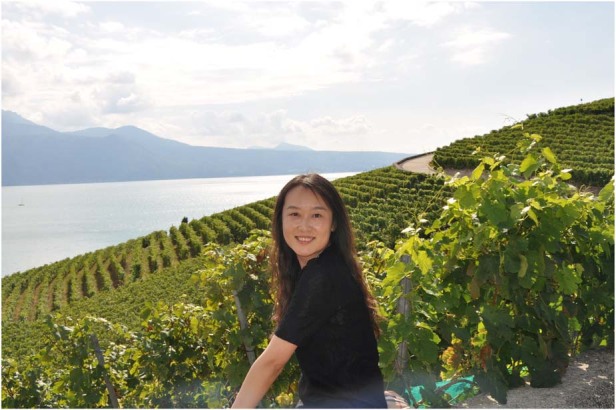


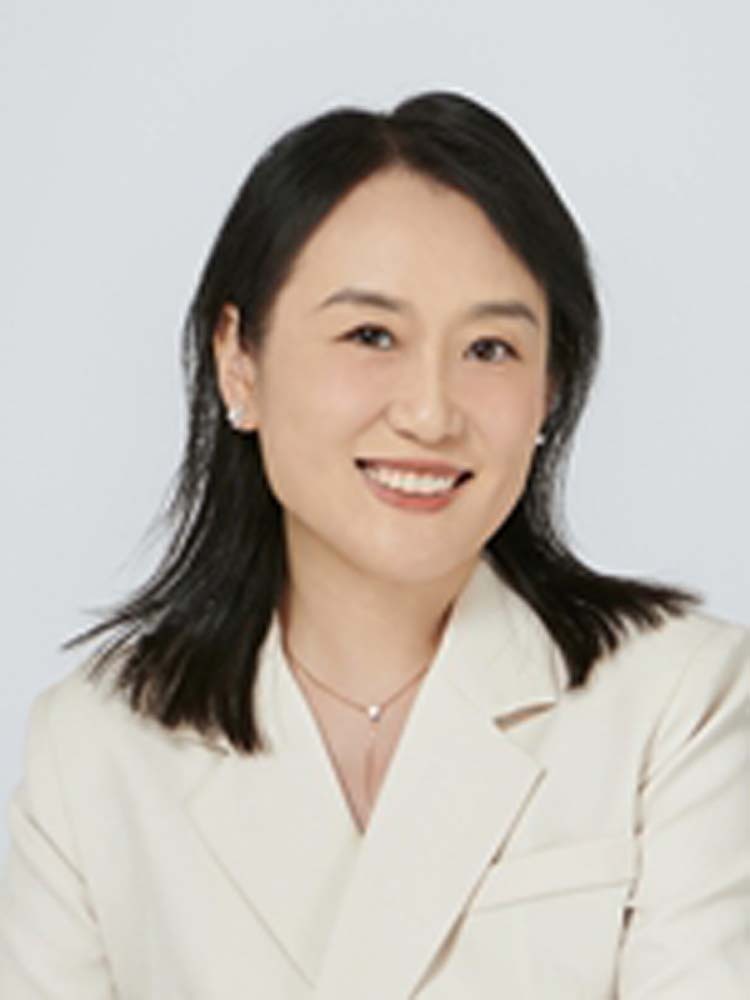
**Biography:** Prof. Laura Na Liu received her Ph.D. in Physics at University of Stuttgart, Germany. She then worked as a postdoctoral fellow at the University of California, Berkeley and as a Texas Instruments visiting professor at Rice University, respectively. Before she became a professor at the Kirchhoff Institute for Physics at University of Heidelberg in 2015, she had worked as an independent group leader at the Max-Planck Institute for Intelligent Systems. In 2020, she joined University of Stuttgart and became the Director of the 2. Physics Institute.

Her research interest is multi-disciplinary. She works at the interface between nanophotonics, biology, and chemistry. Her group focuses on developing sophisticated and smart optical nanosystems for answering structural biology questions as well as catalytic chemistry questions in local environments.

Prof. Laura Na Liu has obtained many prestigious awards, including the Hertha-Sponer Prize (2010), Nanowissenschaftspreis AGENT-D (2011), Sofja Kovalevskaja Award of the Alexander von Humboldt Foundation (2012), Heinz Maier-Leibnitz Prize (2014), European Research Council (ERC) Starting Grant Award (2014), Light 2015 Young Woman in Photonics Award of the European Optical Society (2015), IUPAP Young Scientist Prize in Optics from the International Commission for Optics (2016), the Kavli Foundation Early Career Award in Materials Science (2018), Rudolf-Kaiser Prize (2018), SPIE Rising Researcher Award (2019), Adolph Lomb Medal (2019), The Nano Letters Young Investigator Lectureship Award (2019), EU-40 Materials Prize (2019), Max Planck Fellow (2020), Fellow of Optical Society of America (2020), Fellow of the Royal Society of Chemistry (2023), Fellow of American Physical Society (2023), etc.

**1. Could you briefly introduce your current research direction and main focus please?**

Prof. Laura Na Liu: Currently, we aim to build artificial factories composed of DNA-based cell mimics de novo, which may emulate the designated characteristics of cells with greater freedom but reduced complexity. It may depart from or even possibly outpace the functionality scope of their biological counterparts, for instance, artificial nanosystems with specifically designed optical response and feedback for efficient energy transfer and dissipation. Knowing how to construct artificial factories with fully coordinated components would provide valuable insights into the emergent properties necessary for the origin of life. The adventure of solving the design problems with the help of physical models might also allow us to understand how a cell solves the same issues.

**2. You are not only successful in the field of nanophotonics, but have also worked in the field of structural DNA technology in recent years, demonstrating the broad research prospects of DNA in photonics, storage, biosynthesis, biological motors, etc. When did you first get interested in DNA? What do you think makes DNA so promising?**

Prof. Laura Na Liu: I got interested in DNA technology, when I was a postdoc in Paul Alivisatos’s lab at Berkeley. I realized that DNA nanotechnology could be the ultimate tool to surpass top-down lithography for achieving the 1–10 nm regime accuracy and extending nanophotonic systems to real three dimensions with full optical tunability.

Apart from being a genetic material for inheritance, DNA is an ideal material to build intelligent nanoscale devices. This was the original idea of Nadrian Seeman, the founder of DNA nanotechnology, who hoped to organize biological entities using DNA in high-resolution crystals. Later in 2006, Paul Rothemund attributed to the revolutionary invention of DNA origami. DNA origami offers much higher rigidity than discrete DNA strands. Most importantly, it allows for the organization of individual functional groups on a single template with unprecedented precision, sequence specificity, addressability, and programmability. This allows for the accurate positioning of nanophotonic elements, such as metallic nanocrystals, fluorophores, quantum dots, upconversion nanoparticles on DNA origami and make them move the way one desires via a variety of external means. It thus opens the route towards programmable nanophotonics.

The beauty of DNA nanotechnology is that it stands at the interface among many disciplines. It is an attractive topic, probably because scientists with different backgrounds from physics, chemistry, biology, mathematics, engineering, and materials science can all work on it, applying it as a tool in their research fields.

**Figure:**
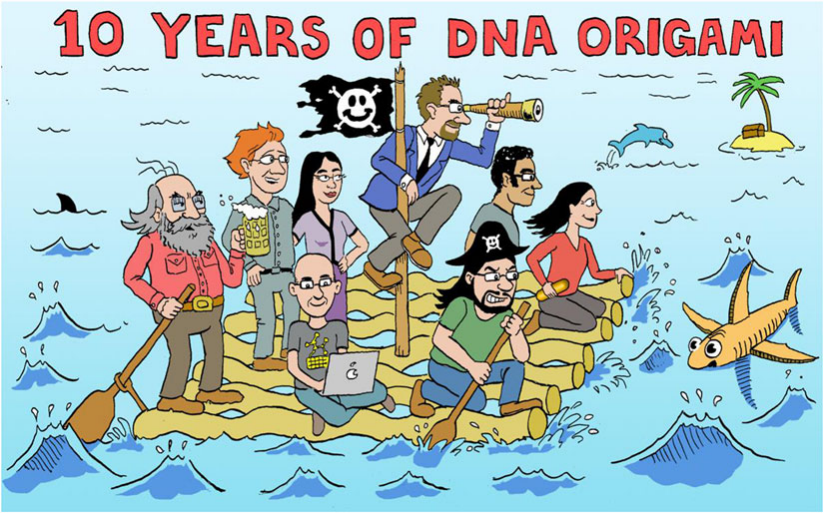
Painting for celebrating the 10^th^ anniversary of DNA origami nanotechnology in 2016. DNA origami nanotechnologists always look forward to new challenges and opportunities. Courtesy of Dr. Ebbe Sloth Andersen

**3. You have conducted basic scientific research on plasmonic nanodevices and DNA structures, what breakthroughs need to take place before these technologies can be industrialized?**

Prof. Laura Na Liu: Outstanding challenges and open questions remain for commercialization and industrialization of DNA-based nanophotonic devices. To name a few, the high cost of DNA synthesis has to be significantly reduced, especially for large and complex DNA structures. Advanced computational tools have to be developed for predicting and optimizing the assembly pathways and outcomes. Most importantly, the switching rate, reversibility, stability, and performance of the dynamic nanophotonic devices have to be substantially enhanced for the needs of practical applications. Although there has been extraordinary research on these challenges, innovative solutions and further investigations await.

**Figure:**
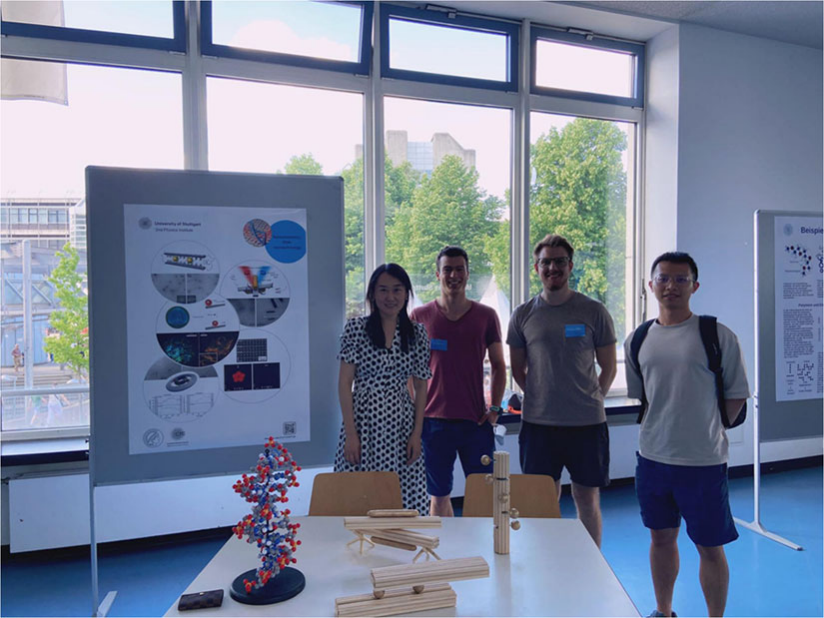
Open science day at University of Stuttgart in 2022

**4. Your research field covers chemistry, biology, nanooptics, etc. How do you feel about interdisciplinary research? How do you discover new research directions?**

Prof. Laura Na Liu: I always believe that the more we understand, the weaker are the boundaries between disciplines. Eventually, different disciplines will converge, because all that we are doing will sought the same end - understanding nature and the universe.

The key to discover new research directions, in my opinion, is the curiosity about things that one still does not understand. When I am at conferences, I often go to sessions that are not in my field. I am interested in what is going on in other research areas, even though I probably will never work exactly on them. However, new ways of thinking and ideas can be inspired from being exposed to completely new knowledge, which is out of one’s comfort zone.

**5. You received the Sofia Kovalevskaya Award for outstanding young foreign scientists from the Humboldt Foundation at the age of 33. It came with a total research grant of 1.5 million euros. How did this award affect your later research career?**

Prof. Laura Na Liu: It was one of the most important turning points in my life. It is also one of the best funding programs I would recommend, if a young researcher, especially an international one, would like to start his/her scientific career in Germany. This program from the Alexander von Humboldt foundation offered me a generous financial support, while my host, the Max Planck Institute eliminated many administrative burdens and provided me an amazing research environment to start, in terms of shared facilities and close collaborations.

**Figure:**
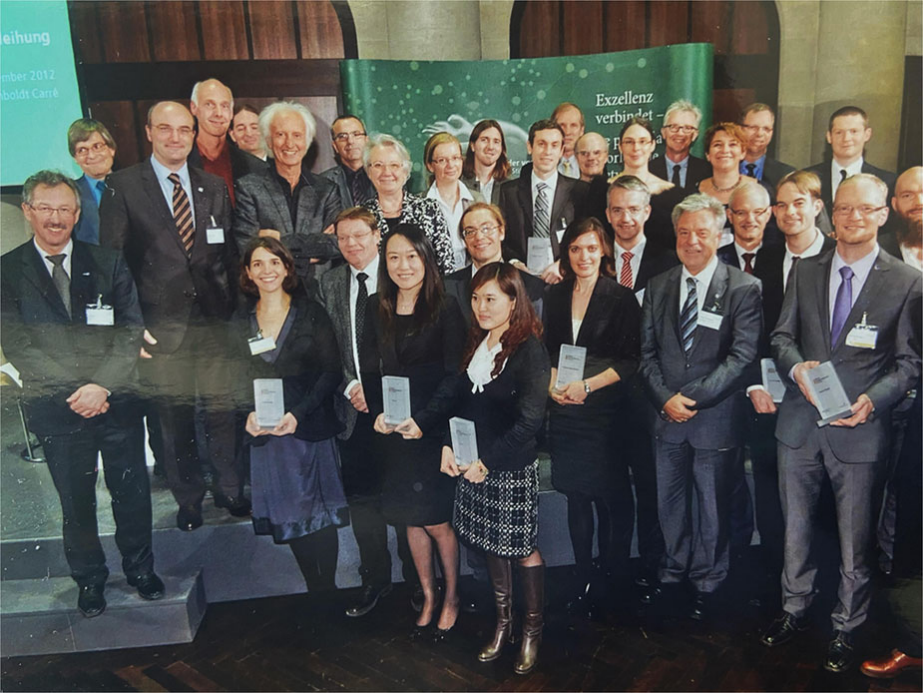
Sofia Kovalevskaya award ceremony in Berlin, 2012

**6. You worked as a tenured professor in the Physics Department of the University of Heidelberg. Why did you join the University of Stuttgart in 2020? How have you changed since joining the University of Stuttgart?**

Prof. Laura Na Liu: The relocation was mainly due to family issues, especially the childcare. When I worked in Heidelberg, I had to commute by train between two cities. It was very challenging, especially when the kid was too little.

**Figure:**
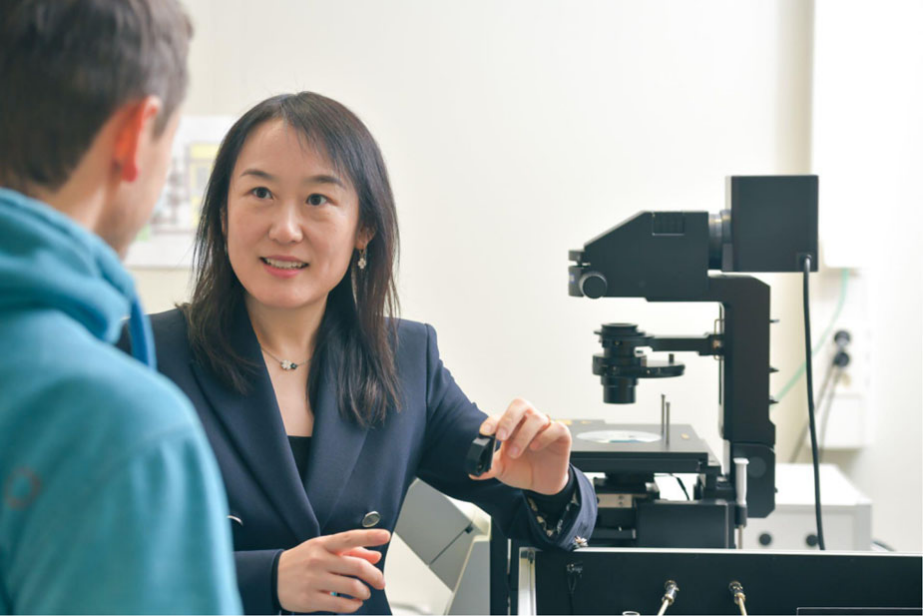
Working in the lab at University of Stuttgart

**7. You are an associate editor of** ***Science Advances*** **and a member of the Editorial Advisory Board for journals such as** ***Nano Letters*** **and others. You have also published more than 100 academic articles in many well-known academic journals (H-index: 62). What do you think make a good academic paper?**

Prof. Laura Na Liu: Well, I am often frustrated by paper submissions, too. I do not know whether I stand at the right position to comment on what makes a good academic paper. However, I am happy to share my suggestions for my students, when they write papers. First, if you believe there is lack of the novelty, please do not start the project. Save energy and funding. Second, after the experiments are completed, make a good figure story and write an attractive abstract. During writing, avoid a storm of hype, as good results speak for themselves. Third, actively communicate with your supervisor during the paper writing to ensure that you stay on the right track. Otherwise, your first draft is likely to be found in his/her trash bin. Also, ask senior labmates and native speakers for help to polish your language and correct errors that you can no longer notice. Then, further polish the paper by your coauthors and supervisor. Finally, submit your paper, and pray it can be sent out and later published.

**8. You were a judge of the “2023 China Academic League of Optics and Optical Engineering Doctoral Students” competition co-sponsored by** ***Light: Science & Applications*, iCANX, and UNESCO “International Day of Light” Organizing Committee. What do you think of this event? Any suggestions for the development of the journal** ***Light: Science & Applications*?**

Prof. Laura Na Liu: It was a great honor to be involved in this event, which offered a fantastic platform for graduate students to present, interact, and learn. I was very grateful for the significant amount of work and effort that the organizers had done to make the event successful. The presentations from the students showed great topic diversity, covering broad expertise in optics and optical engineering. Also, the students were selected from different universities across the country with inherent area diversity. The gender diversity was good, about 20-30% female candidates in total. There is still room to improve, but this is a general issue in our field.

Regarding the journal *Light: Science & Applications*, it is a flagship journal in the field. One of the reasons that the journal could be so successful is attributed to its passionate editors behind the scene, who are engaged in their work and enthusiastic about science. One new exploration for the journal could be setting up a special forum for the LSA authors to share the stories behind their work, including how the original idea was generated, how the collaboration was initiated, what difficulties the authors had to overcome, as well as what depressing and weird data they encountered, etc. This is interesting and insightful for readers, especially for young researchers, who just start their scientific careers.

**Figure:**
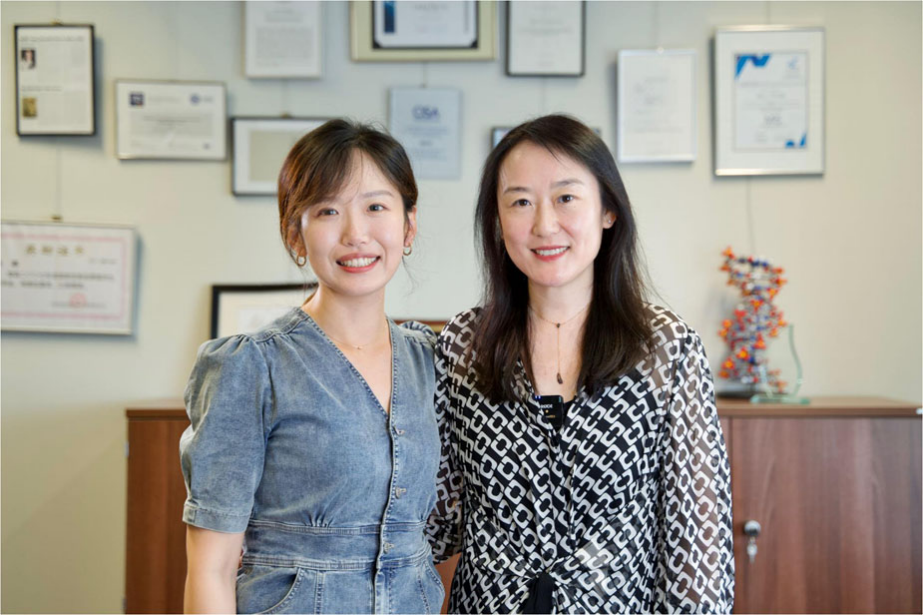
LSA editor, Dr. Jingze Yuan visited Prof. Laura Na Liu’s group in 2022

**9. Could you please share a good research habit of yours?**

Prof. Laura Na Liu: Every two weeks, I collect latest papers that are interesting to me in a literature folder. I then read them collectively (often on Saturday or Sunday evenings). For some papers, I do in-depth reading, while for others I only quickly browse them to grasp the rough ideas.

**10. As a team leader, how do you lead and manage the team?**

Prof. Laura Na Liu: I try to build a collaborative, open, and dynamic group. As the group grows in size, collaborations among group members become more and more crucial. It is about sharing the expertise and taking responsibilities to accomplish common goals together. It is also about technique transfer within the group for continuity and building a good team spirit.

Besides weekly group meetings, we also hold monthly project meetings within subgroups for intense discussions, especially on difficult and unexpected experimental results. I encourage my group members to apply for individual grants/funding, so that they acquire skills on logic thinking, proposal writing, and interviews. I also send my students to many international conferences, so that they learn how to improve their presentation skills and become confident to speak in front of large audience. Most importantly, they get to know their peers and established researchers already at a young age for networking and future collaborations.

**Figure:**
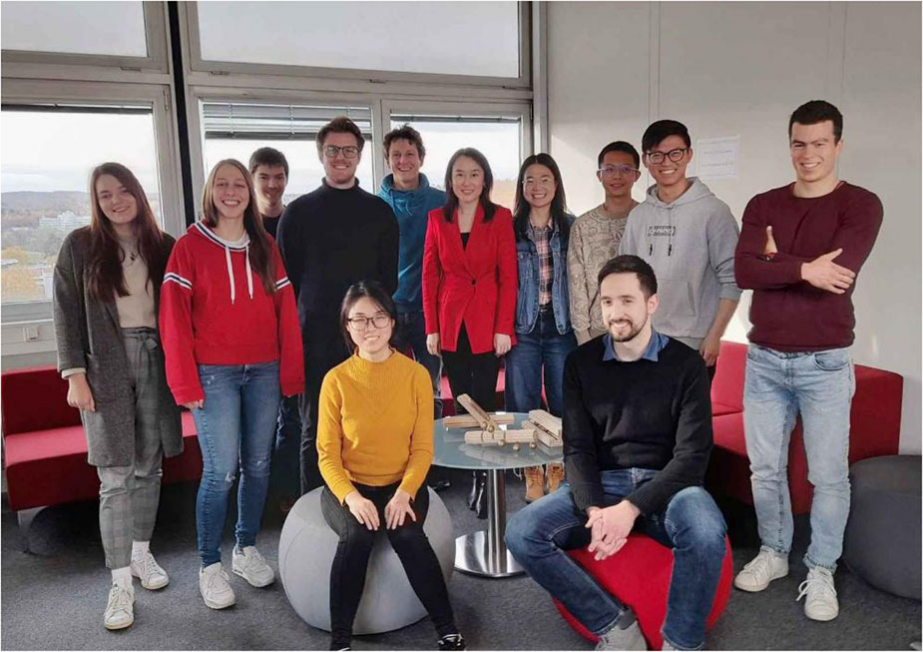
Group photo in 2022

**11. What do you think is the biggest gain in your work?**

Prof. Laura Na Liu: I get more self-disciplined and goal oriented through my work. In order to achieve a better balance between work and life (probably there is never a real balance between them for a professor), I have to make plans for many things, such as project meetings, institute matters, faculty meetings, teaching, traveling, childcare, etc. Self-discipline is the solution to multi-tasking. I am still an old-styled person. I do not use Google calendar. I have a book planner, on which I write all the daily tasks and plans.

**12. On March** **8**^**th**^ **this year, you participated in the Rose in Science event sponsored by Changchun Institute of Optics, Fine Mechanics and Physics (CIOMP), Chinese Academy of Science (CAS) and co-organized with iCANX. What was the experience like for you? Do you have any suggestions for improvements?**

Prof. Laura Na Liu: It was a journey to learn, support, and share with other female scientists. Due to Covid-19, it took place online. It will be great, if this event can take place on site with young female students as audience, so that they can directly participate and interact with the speakers.

**Figure:**
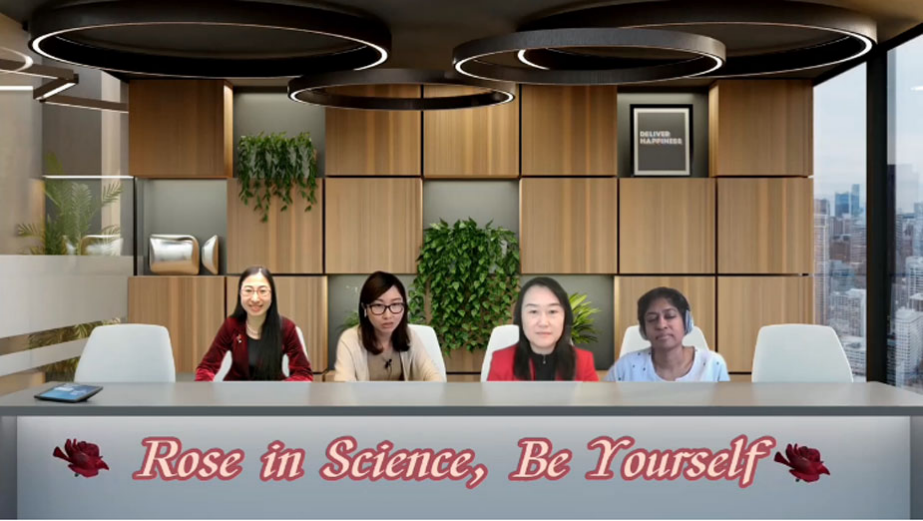
Prof. Laura Na Liu attended the 2023 Rose in Science Event

**13. What have been the biggest difficulty or challenge you have encountered in your career? How did you conquer it?**

Prof. Laura Na Liu: Having a child was the biggest change to my life, which was challenging at the beginning. Work started to be piled up - unanswered emails, losing track of project progress, declining conference invitations, and even no time to be sad about the rejections of my papers and funding applications. There was a lot of frustration, until one day my kid pointed at one picture in her book and said ‘it is mom. I took a close look at that picture. It was a lady, who sat next to a table. She was reading a book, while she put her hand on her forehead, showing a grumpy and exhausting face. I was shocked to realize that I had turned myself into a very unhappy person and this was the way my kid thought about me. I also realized that it was a mistake to mix childcaring and working times together, such that neither of them was done well. At that point, I decided to make a change. The solution was to have a clear cut between work and childcare. When I spend time with my kid, I try not to think about my work. When I am at work, I fully concentrate on it. Even though often I still end up my day with a lot of unfinished work, I no longer feel bad about myself and accept the fact that this is the new phase of my life.

**Figure:**
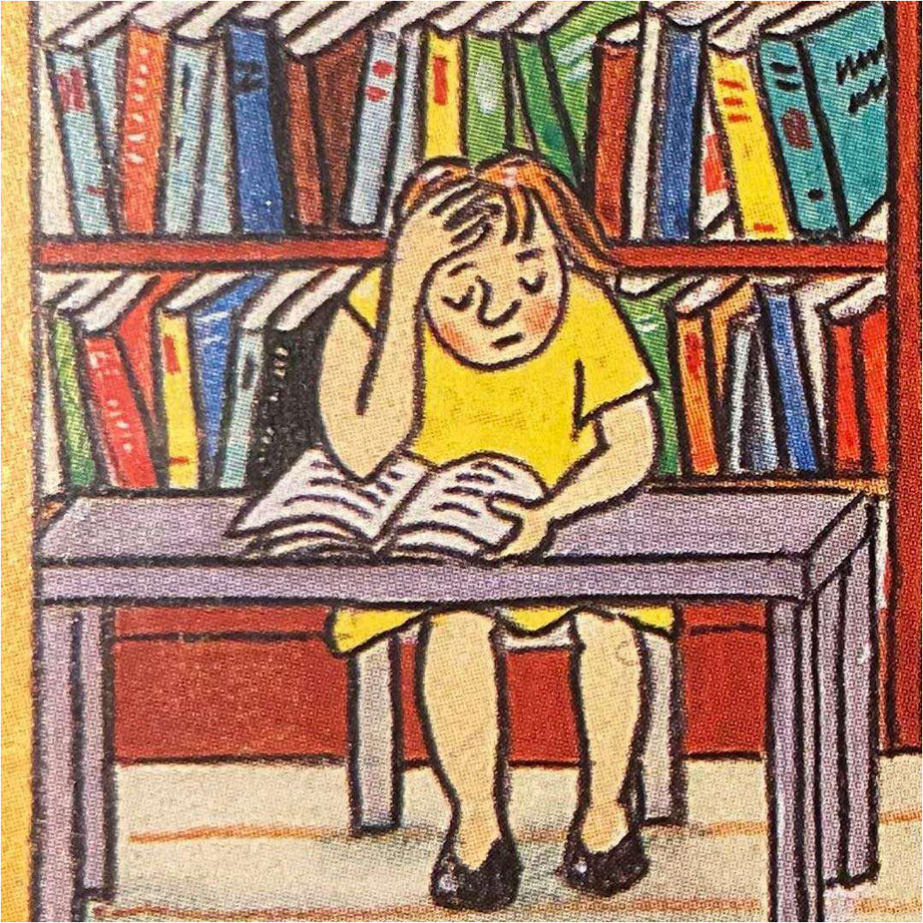
The picture from a book owned by Prof Laura Na Liu’s child

**14. Who would be your science hero?**

Prof. Laura Na Liu: I do not have a defined science hero, as there are many great people to admire. In the last two years, I have been teaching Advanced Condensed Matter Physics in our department. It was truly exciting to show the students the ground-breaking works of the great minds and their stories behind, and most importantly on what bases they had developed those physics models. What a golden age of physics back then! Imagine, at the Solvay conference, how sensational it should have been that the greatest physicists of all time, such as Einstein, Bohr, Dirac, Marie Curie, Schrödinger, among others, could all sit together and devote to pre-eminent open questions in physics. So, are we now at a new golden era of physics?

**15. What are your hobbies?**

Prof. Laura Na Liu: I love traveling to different places for experiencing different cultures, people, and food. My current hobbies are building sandcastles and gardening with my daughter.

**Figure:**
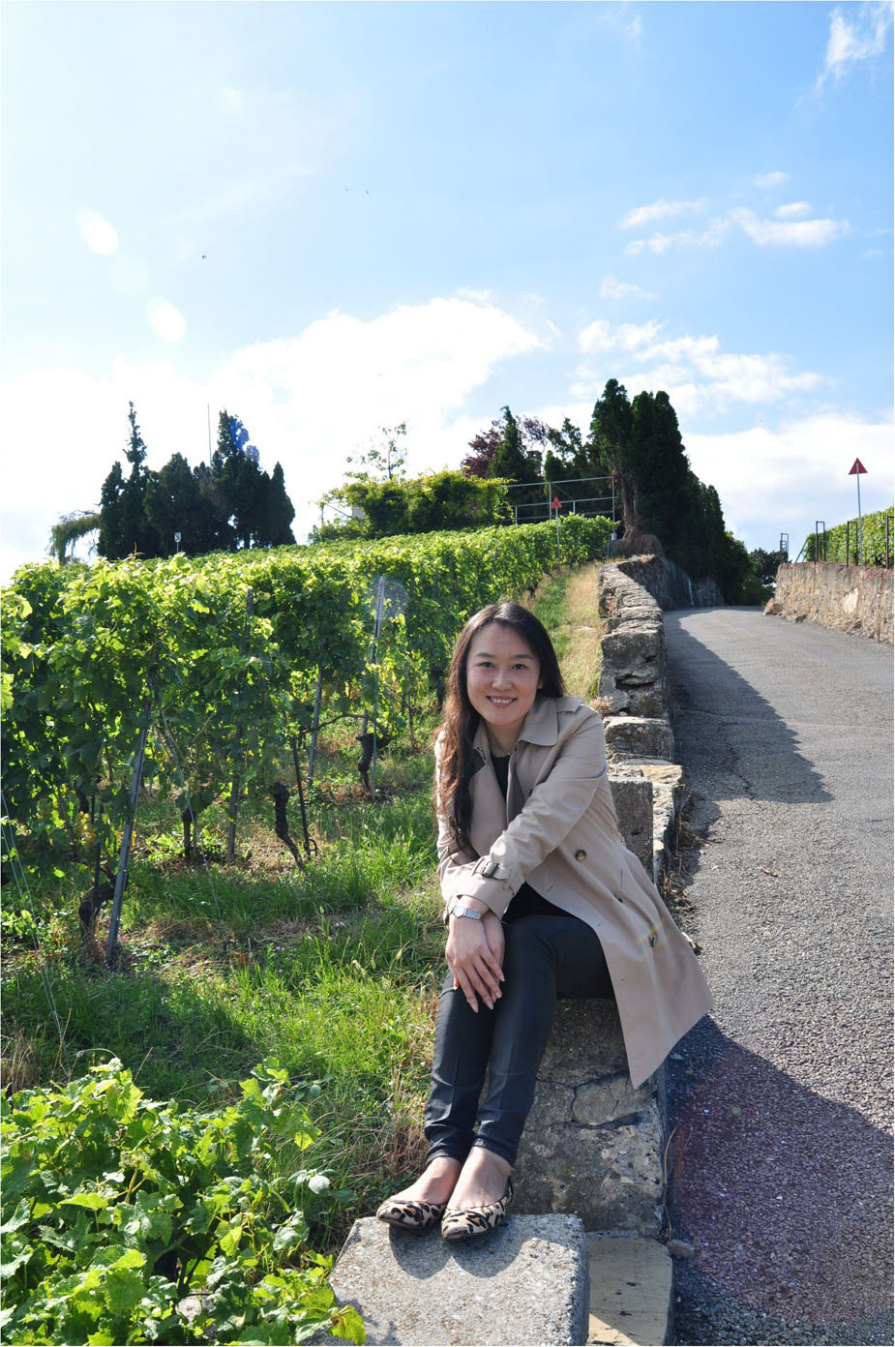
Visiting a vineyard in Switzerland

**16. What advice and suggestions would you like to give young researchers?**

Prof. Laura Na Liu: When I was young, I was never the smartest student in my class, but I was always the most persistent kid. I believe ‘shoot for the moon! Even if you miss, you’ll land among the stars’. So be persistent and patient, as there is always a second chance in life.

